# Robust federated learning for UAV object detection: a joint self-distillation and drift compensation approach

**DOI:** 10.3389/fnbot.2026.1649168

**Published:** 2026-04-01

**Authors:** Yu Hangsun, Changnan Jiang, Ziyuan Zhang, Heqing Ouyang, Pengpeng Chen

**Affiliations:** 1 School of Communication and Information Engineering, Nanjing University of Posts and Telecommunications, Nanjing, China; 2 Beihang University, Beijing, China; 3 University of Toronto, Beijing, China; 4 The University of Hong Kong, Hong Kong, China

**Keywords:** data heterogeneity, federated learning, model drift, self-distillation, UAV object detection

## Abstract

The rapid advancement of unmanned aerial vehicles (UAVs) in disaster response and environmental monitoring has underscored the growing importance of real-time object detection within UAV swarm networks. However, the non-independent and identically distributed (non-IID) characteristics of data in UAV networks present significant challenges to model convergence and adaptability. To tackle these challenges, this study introduces a robust federated UAV object detection framework tailored for non-IID data distributions. The framework aims to enhance adaptability across clients, thereby improving both detection performance and convergence speed. Our approach includes a self-distillation mechanism that leverages personalized knowledge from local model historical states to guide current local training, striking a balance between specialization and adaptability. Additionally, we propose a drift compensation mechanism to synchronize local and global model updates, mitigating model drift. We conducted extensive experiments on the VisDrone2019-DET dataset, comparing our method to baseline models. Results demonstrate that our approach accelerates convergence speed by approximately 2.2 times and enhances detection performance by around 3%, offering an efficient and robust solution for UAV-based object detection under non-IID conditions.

## 1 Introduction

Unmanned aerial vehicles (UAVs) have experienced rapid expansion across various domains, including disaster response, environmental surveillance, and intelligent transportation systems. The efficacy and performance of these applications hinge significantly on real time object detection. These applications demand real-time object detection that can adapt to diverse and dynamic environments. However, deploying object detection on UAVs faces distinct challenges at different stages: at the inference stage, limited computational resources necessitate lightweight models; at the training stage, when multiple UAVs collaborate, centralized data aggregation raises privacy concerns and bandwidth limitations. Recent strides in deep learning have markedly enhanced object detection accuracy in UAV imagery, with the advent of lightweight convolutional approaches enabling the practical implementation of these models on resource constrained UAV platforms (Zhu et al., 2021a; Wu et al., 2024). Nevertheless, these methodologies often necessitate centralized data aggregation, posing privacy vulnerabilities and proving unsuitable for decentralized frameworks like multi UAV networks (Hafeez et al., 2024). Recent surveys and state of the art detection models further illustrate both progress and challenges in UAV based visual perception. A comprehensive survey of deep learning methods for UAV object detection and tracking highlights challenges in small object detection and complex backgrounds (Wu et al., 2021). An overview of emerging deep learning techniques for UAV imagery emphasizes dense small object scenarios and adaptation to onboard platforms (Tang et al., 2023).

Federated Learning (FL) has emerged as a prominent method for preserving privacy, attracting considerable attention in research by facilitating collaborative model training across decentralized devices without the need to share raw data (Liu et al., 2021). This approach holds significant promise for applications involving cooperation among multiple Unmanned Aerial Vehicles. However, UAVs are commonly deployed in diverse environments characterized by variations in object scales, lighting conditions, and scene complexity, factors that can impede model convergence and adaptability (Nguyen et al., 2021). The resulting data heterogeneity leads to inconsistent local updates and unreliable global aggregation, presenting substantial challenges for the practical implementation of FL in UAV systems. Consequently, FL encounters significant hurdles in tasks such as object detection on UAV platforms. To address these deployment and heterogeneity issues, recent FL solutions such as FedYolo (Zhang et al., 2023) and ACSFed (Chen et al., 2022) demonstrate enhanced convergence and resilience in non-IID environments. FedYolo and ACSFed have been incorporated into our baseline comparison to provide a more comprehensive analysis. FedYolo modifies the YOLO detection head for federated scenarios using transformer based attention to address client drift. On the other hand, ACSFed utilizes an adaptive client selection scheme that selects participants dynamically based on divergence and reliability.

Various strategies have been developed to address the challenges faced by Federated Learning in complex visual tasks, such as data heterogeneity and model performance issues (Fu et al., 2022; Zhagypar et al., 2022). One such strategy is semi-supervised optimization, which leverages unlabeled data more effectively in federated settings, thereby enhancing model performance in scenarios with limited data availability (Zhang et al., 2022). Another line of work, exemplified by AutoFed (Zheng et al., 2023), introduces heterogeneity aware multimodal learning to improve robustness and adaptability in non-IID environments. Additionally, approaches like asynchronous updates and differential privacy mechanisms have been suggested to improve system robustness against client staleness and safeguard data privacy during transmission (Pan et al., 2023). Nevertheless, these methods do not fully tackle critical issues like local overfitting, personalized knowledge forgetting, and model drift resulting from data heterogeneity. These challenges underscore the necessity for more resilient solutions in federated UAV-based object detection within non-IID environments.

In this study, we introduce a framework termed FL-JSDDC (Federated Learning with Joint Self-Distillation and Drift Compensation) to tackle the challenges arising from non-IID data in federated UAV object detection. Our approach addresses two key aspects. Firstly, at the local model training level, we incorporate a self-distillation mechanism to improve the adaptability of local models and preserve their ability to adapt to client-specific data. Secondly, at the global model aggregation stage, we propose a drift compensation mechanism to alleviate model drift resulting from data heterogeneity. By integrating these two perspectives, we establish a more resilient and effective framework for federated UAV object detection. Our contributions can be succinctly outlined as follows:

- We present FL-JSDDC, a new federated UAV object detection framework that integrates self-distillation and drift compensation mechanisms to address the complexities arising from non-independent and identically distributed (non-IID) data in federated UAV object detection. Our method significantly improves the resilience and detection accuracy of federated UAV object detection models in diverse environments.
- We introduce the self-distillation algorithm to enhance the adaptability of local UAV models by enabling the assimilation of personalized knowledge from past local models. Additionally, we present the drift compensation algorithm, which reduces model drift by dynamically aligning local model updates with the global model. This approach aims to improve model convergence and enhance detection performance in non-IID scenarios.
- Extensive experiments were carried out on the VisDrone2019-DET dataset to compare FL-JSDDC with multiple baseline models under both IID and non-IID conditions. The findings indicate that FL-JSDDC exhibits notable superiority over current FL methods, manifesting enhanced convergence speed and detection performance, particularly in scenarios characterized by data heterogeneity.

## 2 Related work

### 2.1 UAV-based object detection

The rapid progress in UAV technology has led to their growing utilization across diverse fields including environmental monitoring, disaster response, and intelligent transportation. Due to their adaptability and aerial vantage point, UAVs are well-suited for object detection tasks. Nevertheless, achieving precise and timely object detection in intricate settings poses a notable obstacle in the realm of computer vision.

Recent advancements in deep learning have greatly improved the ability of drones to detect objects. The TPH-YOLOv5 model integrates a Transformer Prediction Head into the YOLOv5 framework to enhance detection accuracy across various object scales. Additionally, it incorporates a Convolutional Block Attention Module (CBAM) to improve detection performance in crowded environments, as illustrated in the study by Zhu et al. (2021b). To improve computational efficiency, a selective image patch processing approach based on convolutional neural networks was proposed by Plastiras et al. (2019). By employing attention mechanisms to selectively target object-containing regions, this technique streamlines computation and enhances detection speed without compromising detection accuracy, rendering it particularly suitable for UAV applications with limited resources. Wu et al. (2017) introduced a vision-based system integrating object detection with Kalman filter-based tracking. This methodology facilitates real-time localization and tracking of aerial targets during UAV missions, demonstrating robustness and responsiveness across diverse environmental settings. A comprehensive survey was conducted by Wu et al. (2021) to provide an extensive overview of UAV-based object detection and tracking. The study explores the utilization of deep learning in UAV imagery and video analytics, identifies current challenges, and suggests potential research avenues for the future.

Regrettably, despite these advancements, current methods encounter notable challenges in the practical deployment of UAVs. Many existing approaches depend on centralized data collection, leading to privacy issues and impracticality in distributed UAV networks.

### 2.2 Federated learning-based object detection

The progress of Federated Learning (FL) has led to its increasing utilization in image recognition and object detection, especially in sensitive contexts like multi UAV collaboration, traffic surveillance, and edge computing. FL facilitates collaborative model training among multiple clients without the need to exchange raw data, effectively addressing issues related to data silos and privacy breaches.

Recent studies have advanced the field by investigating privacy, heterogeneity, and drift resilience in federated object detection. For instance, Wang et al. (2024) proposed a federated object detection framework incorporating dynamic differential privacy. This approach adjusts noise levels based on feature sensitivity and training progress, achieving competitive detection accuracy on COCO and PASCAL VOC benchmarks while ensuring privacy. Similarly, Rashidi et al. (2024) introduced a self-configuring federated medical object detection framework that can adapt aggregation strategies to diverse and non-IID medical imaging data. Their work demonstrated feasibility on radiology datasets from multiple institutions. Furthermore, Awaysheh et al. (2024) conducted an empirical investigation on detecting drift in federated learning. Their study revealed that even minor concept or data drift at a single client can significantly impair the overall model performance, highlighting the critical need for robust drift-aware mechanisms.

The study by Zhang et al. (2022) introduces a robust semi-supervised federated learning (SSFL) framework tailored for UAV-based image recognition tasks. The framework proposes a FedMix parameter mixing strategy to facilitate model transfer between the server and clients. It also incorporates a FedFreq aggregation rule to dynamically adjust client weights, thereby enhancing model detection performance under non-IID data distributions. This approach effectively balances privacy preservation and model accuracy, showcasing the potential of SSFL in aerial image analysis. In a real-time scenario, Xie et al. (2023) present an asynchronous federated learning (AFL) framework for multi-license plate recognition. This method combines semantic communication with multi-task learning (MTL), formulating the problem as a multi-objective optimization task. The optimization is solved using the multi-gradient descent algorithm (MGDA). The asynchronous strategy proposed addresses client staleness during training, thereby improving system robustness and communication efficiency. Moreover, to enhance multi-task recognition while ensuring privacy, Xie et al. (2024) introduce a differentially private federated multi-task learning framework (DP-FL). This framework formulates object recognition as a multi-objective task and integrates local differential privacy by injecting calibrated noise into local gradients. Experimental results demonstrate that the method achieves strong detection performance and privacy protection across various benchmark datasets. Khazaei et al. (2025) introduced an optimal server representation (OSR) based Federated Learning (FL) framework to enhance training efficiency and minimize communication overhead. This approach involves sharing a limited set of privacy-preserving representative samples instead of transmitting model parameters to reduce bandwidth usage. Additionally, the framework incorporates knowledge distillation to assist local models in acquiring global representations, consequently enhancing recognition accuracy in visual tasks.

Despite recent advancements, current methods face significant challenges. While privacy and communication efficiency have been studied, ensuring consistent model accuracy across heterogeneous data distributions remains a critical hurdle due to the non-IID nature of data across clients. These challenges underscore the necessity for more robust and precise solutions in real-world federated UAV object detection scenarios.

## 3 System model and problem description

In this section, we introduce the system model, problem description and design objective considered in this work.

### 3.1 System model

#### 3.1.1 System architecture

We consider a federated UAV object detection framework consisting of a central server (base station) and multiple UAV clients. Each UAV is equipped with a certain amount of onboard image data and computational resources. The server coordinates the UAVs to collaboratively build the federated object detection model. Without sharing raw data, the system performs global aggregation to generate a high quality global object detection model.

**Figure 1:**
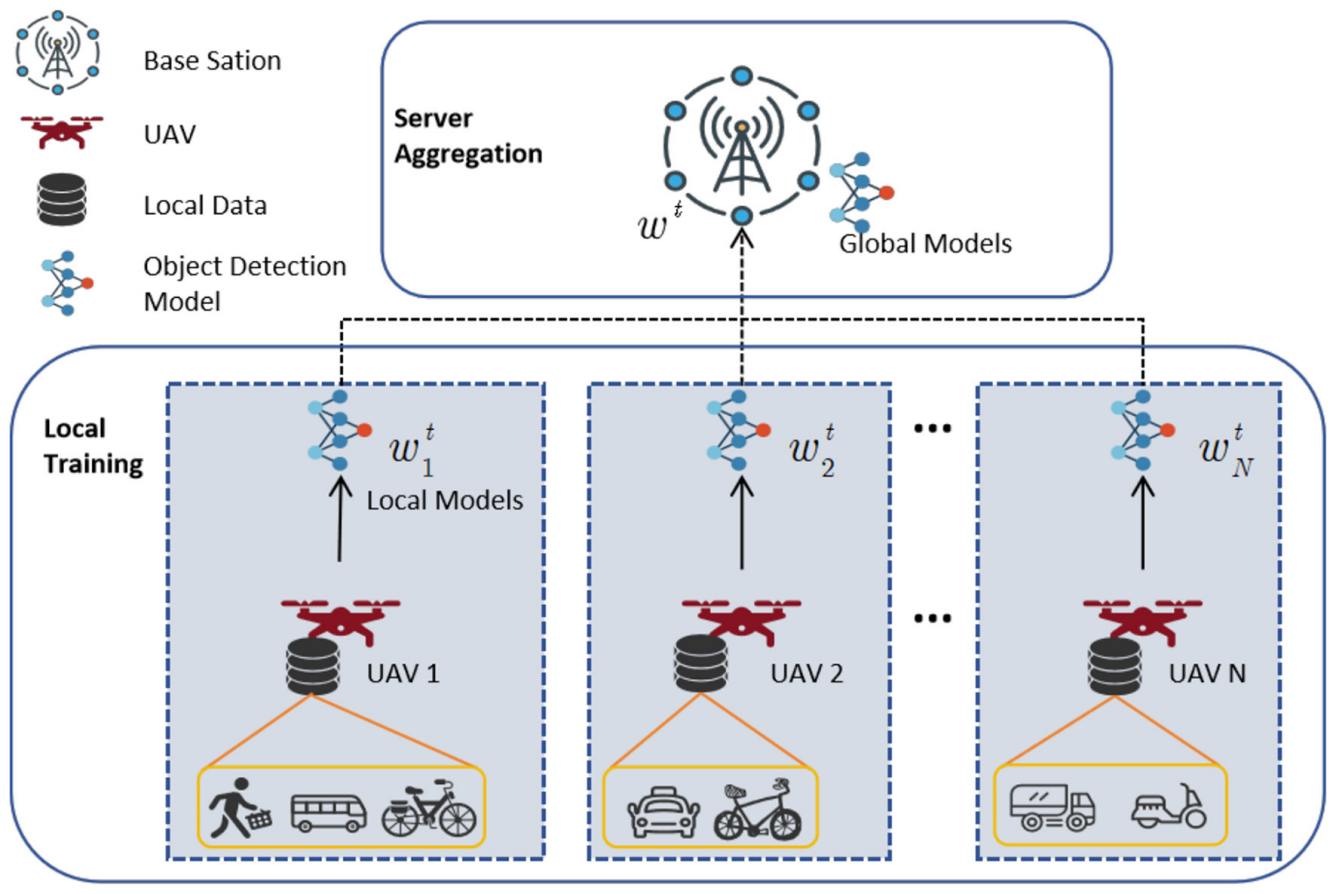
Scenario diagram of a federated UAV object detection framework.

The system, depicted in Figure 1, comprises a central server (base station) and multiple clients (UAVs). In each communication round, individual UAV clients train a local object detection model using onboard visual data and transmit the updated model parameters to the central server. Upon receiving the local models from all clients, the server aggregates them to update a global model, which is then disseminated to all UAVs for subsequent training rounds. This decentralized learning approach eliminates the necessity to exchange raw data, thereby safeguarding privacy. Let *S* represent the central server, and let the *N* participating UAVs be denoted as distributed clients within the system. Each client *i* possesses a local dataset *D*_*i*_, with data distributions among clients typically being non-IID. Local training is conducted exclusively based on the dataset of each client.

#### 3.1.2 Local training objective

The objective of each client *i* is to minimize the following local loss function during on device training:


$$\begin{array}{l} F_{i}\left(w_{i}\right)=\frac{1}{\left|D_{i}\right|}\underset{\left(x_{j},y_{j}\right)\in D_{i}}{\sum }\zeta_{YOLO}\left(w_{i};x_{j},y_{j}\right)+\beta R\left(w_{i}\right) \end{array}$$ (1)

where $w_{i}\in {\mathbb{R}}^{d}$ denotes the local model parameters of client *i*, and *D*_*i*_ represents the local dataset. Here, *x*_*j*_ is the input image and *y*_*j*_ is the corresponding annotated label including bounding boxes and class information. The term *R*(*w*_*i*_) is a regularization function, such as L2 regularization, and β is a nonnegative scalar that controls the regularization strength. The function ζ_*YOLO*_ denotes the YOLOv8 object detection loss, which typically consists of three components: classification loss, objectness confidence loss, and bounding box regression loss.


$$\begin{array}{l} \zeta_{YOLO}\left(w_{i};x_{j},y_{j}\right)=L_{cls}+L_{obj}+L_{box} \end{array}$$ (2)

Specifically, the classification loss, objectness confidence loss, and bounding box regression loss are defined as follows:


$$\{\begin{array}{l} {ℒ}_{cls}=-\sum_{c=1}^{C}y_{j}^{\left(c\right)}\log({\hat{q}}_{j}^{\left(c\right)})\\ {ℒ}_{obj}=-\left[y_{j}^{obj}\log({\hat{q}}_{j}^{obj})+(1-y_{j}^{obj})\log(1-{\hat{q}}_{j}^{obj})\right]\\ {ℒ}_{box}=1-\frac{A_{int}}{A_{uni}} \end{array}$$ (3)

where *C* is the number of object categories, $y_{j}^{\left(c\right)}$ is the ground truth label for class *c*, and ${\hat{q}}_{j}^{\left(c\right)}$ is the predicted class probability. $y_{j}^{obj}\in \left\{0,1\right\}$ indicates the presence of an object, and ${\hat{q}}_{j}^{obj}$ is the predicted objectness score. *A*_*int*_ denotes the intersection area between the predicted and ground truth bounding boxes, and *A*_*uni*_ denotes their union area.

#### 3.1.3 Global aggregation objective

After each communication round, the central server collects updated local model parameters from all clients and aggregates them to update the global model using a weighted averaging strategy: $w=\overset{N}{\underset{i=1}{\sum }}p_{i}w_{i}$, where *w* denotes the global model parameters, and the weight *p*_*i*_ is determined by the relative size of each client's local dataset:$p_{i}=\frac{\left|D_{i}\right|}{\overset{N}{\underset{n=1}{\sum }}|D_{n}|}$, the overall optimization objective of the system is to minimize the global empirical risk:


$$\begin{array}{l} \underset{w}{\min}F\left(w\right)=\overset{N}{\underset{i=1}{\sum }}p_{i}F_{i}\left(w\right) \end{array}$$ (4)

where *F*_*i*_(*w*) represents the local loss function of client *i* evaluated on the global model *w*. This objective ensures that the aggregated model is jointly optimized across all clients, with contributions weighted by data volume, thereby producing a globally shared model that strikes the best compromise in applicability across all clients.

### 3.2 Problem description

Although federated UAV-based object detection systems have shown great potential in distributed collaborative training and privacy preserving applications, their practical deployment, especially for complex tasks such as object detection, still faces significant challenges. One of the most critical issues is the detection performance degradation and convergence difficulty caused by data heterogeneity, that is, non-IID data across clients.

In a conventional federated detection framework, it is typically assumed that the data distributions across clients are similar. In practical federated UAV object detection scenarios, this assumption often does not hold. The local data distribution *P*_*i*_(*x, y*) held by client *k* often differs significantly from the global data distribution. Taking input samples *x* and predicted class labels *y* as an example, the local training process typically aims to minimize the cross entropy loss to maximize the conditional probability *P*(*y*|*x*), i.e.,


$$\begin{array}{l} \underset{w_{i}}{\min}E_{\left(x,y\right)\sim D_{i}}\left[-\log P\left(y|x;w_{k}\right)\right] \end{array}$$ (5)

According to Bayes' theorem, the conditional probability *P*(*y*|*x*) can be rewritten as:


$$\begin{array}{l} P\left(y|x\right)=\frac{P\left(x|y\right)P\left(y\right)}{P\left(x\right)} \end{array}$$ (6)

where *P*(*x*|*y*) is the class conditional distribution, *P*(*y*) is the class prior distribution, and *P*(*x*) is the marginal distribution of input features. This decomposition shows that local model optimization is influenced not only by the conditional distribution *P*(*x*|*y*) but also by the class prior *P*(*y*).

However, in practical federated UAV object detection systems, heterogeneity in client data, caused by variations in sensing devices, environments, and application scenarios, leads to shifts in both *P*(*x*|*y*) and *P*(*y*). These shifts cause local models to overfit to client data distributions and converge toward different optima, which leads to inconsistent updates and degraded global detection performance during aggregation.

### 3.3 Design objectives

To address the detection performance degradation caused by data heterogeneity in federated UAV object detection framework, this paper proposes an improved training mechanism based on the idea of optimizing local adaptability and global model consistency. This mechanism incorporates self-distillation and drift compensation to mitigate the impact of model drift, enhance robustness and adaptability, and thereby accelerate convergence and improve detection performance.

## 4 Methodology

### 4.1 Proposed method

To address the challenges of data heterogeneity and model drift in federated UAV object detection, we propose a novel method named **FL-JSDDC**. Built upon the YOLOv8n detection backbone, FL-JSDDC combines local knowledge transfer and global model alignment strategies to improve detection performance and convergence speed under non-IID distributions.

**Figure 2:**
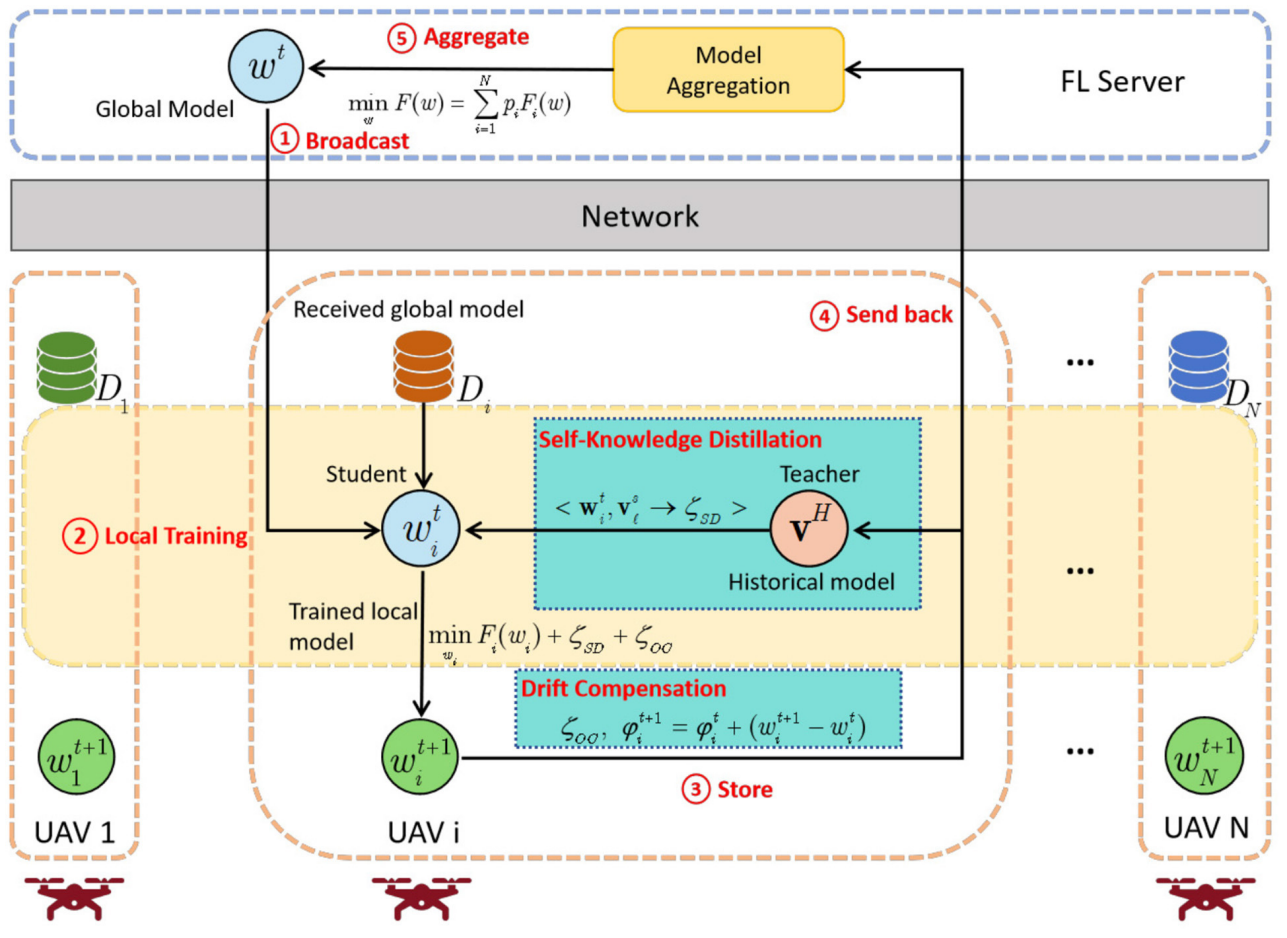
Overview of the FL-JSDDC model.

The overall framework is illustrated in Figure 2, and consists of the following two core modules:

**(1) Self-distillation mechanism:** This module leverages personalized knowledge from the historical local models to guide current training. It preserves client specific representations and mitigates local forgetting by aligning past knowledge with current updates.
**(2) Drift compensation mechanism:** This module reduces global model drift caused by non-IID data by explicitly estimating and compensating for the deviation between local and global models. It helps align model updates across clients for more stable aggregation.

The overall process of FL-JSDDC proceeds in the following five steps:

**(1) Local training:** Each client initializes its local model with the global model and performs training on its local dataset.
**(2) Self-distillation mechanism:** During training, each client employs a self-distillation mechanism that uses historical local models to generate soft targets, helping retain personalized knowledge and guide current training.
**(3) Drift compensation mechanism:** Upon completing training, each client computes an drift vector between the updated local model and its historical local model, to compensate for model drift caused by non-iid data.
**(4) Model aggregation:** The compensated local models are uploaded to the server. The server aggregates them to produce a globally consistent model.
**(5) Model broadcast:** The updated global model is then sent back to all clients, initiating the next round of federated training.

In each communication round, the self-distillation mechanism first enhances local adaptability by leveraging historical knowledge, followed by the drift compensation mechanism that aligns local updates with the global objective, improvements are made from both local training and global aggregation perspectives to ensure robust across non-IID data environments.

#### 4.1.1 Self-distillation mechanism

We introduce a supervised self-distillation framework for individual client detectors, comprising five consecutive stages: an input block, a bounding box alignment block, a global class probability alignment block, a channel semantic alignment block, and an output block. Raw images remain within the UAV client and knowledge transfer occurs from both the historical personalized local model and the global model (teacher) to the local model (student) through the three alignment blocks delineated below.

**(1) Input block** After standard preprocessing, each minibatch is forwarded through both the historical local model (teacher) and the current local model (student) via parallel forward propagation. The historical local model is retained from previous communication rounds and serves as a personalized knowledge reservoir. This process yields corresponding classification logits $\left\{{\text{z}}_{T}^{\left(j\right)}\right\},\left\{{\text{z}}_{S}^{\left(j\right)}\right\}\in {\mathbb{R}}^{C}$ for each detection token *j* = 1, …, *M*, which are used in the global class probability alignment loss ζ_*G*_ in Equation 8. Simultaneously, it extracts intermediate feature maps $\left\{F_{\ell }^{T}\right\},\left\{F_{\ell }^{S}\right\}$ from head layer ℓ, where $F_{\ell }\in {\mathbb{R}}^{C_{\ell }\times H_{\ell }\times W_{\ell }}$ denotes the activation tensor at head layer ℓ; Together, the generated outputs including classification logits, objectness scores, and spatial features form the basis for subsequent knowledge alignment.

**(2) Bounding Box Alignment block** To distill localization knowledge, we align the regression predictions between the teacher and student models. Specifically, let the predicted normalized bounding box coordinates for each detection token *j* be denoted as $b_{T}^{\left(j\right)}=\left[x_{T}^{\left(j\right)},y_{T}^{\left(j\right)},w_{T}^{\left(j\right)},h_{T}^{\left(j\right)}\right]$ and $b_{S}^{\left(j\right)}=\left[x_{S}^{\left(j\right)},y_{S}^{\left(j\right)},w_{S}^{\left(j\right)},h_{S}^{\left(j\right)}\right]$ for the teacher and student models, respectively. The bounding box alignment loss is computed as:


$$\begin{array}{l} \zeta_{box}=\frac{1}{M}\overset{M}{\underset{j=1}{\sum }}||b_{T}^{\left(j\right)}-b_{S}^{\left(j\right)}|{|}_{2}^{2} \end{array}$$ (7)

where ||·||_2_ denotes the ℓ_2_ norm. The vector *b*^(*j*)^∈ℝ^4^ contains the center coordinates (*x*, *y*), width *w*, and height *h* of the bounding box for token *j*.

This loss encourages the student model to produce bounding boxes that are spatially consistent with the teacher's regression outputs, thereby improving localization accuracy under non-IID data distributions.

**(3) Global class-probability alignment block** Let ${\text{z}}_{T}^{\left(j\right)},{\text{z}}_{S}^{\left(j\right)}\in {\mathbb{R}}^{C}$ denote the teacher and student classification logits of the *j*-th detection token. Following temperature scaling, the alignment loss is as shown in Equation 8.


$$\begin{array}{l} \zeta_{G}=\frac{1}{M}\overset{M}{\underset{j=1}{\sum }}\mathrm{KL}\left(\sigma \left({\text{z}}_{T}^{\left(j\right)}/\tau_{G}\right)∥\sigma \left({\text{z}}_{S}^{\left(j\right)}/\tau_{G}\right)\right) \end{array}$$ (8)

where σ(·) is the sigmoid (multi-label) or softmax (single-label) function, *C* is the number of classes, τ_*G*_>0 is the temperature, and KL(·∥·) denotes the Kullback-Leibler divergence. Minimizing Equation 8 transfers high-level semantic priors from teacher to student.

**(4) Channel-semantic alignment block** To further improve spatial awareness, we align the relative channel importances between teacher and student over head layer ℓ (including three feature maps P3, P4, and P5). This process consists of two steps: extracting spatial channel means and then computing a KL divergence over their softmax scaled distributions.

To align the channel semantics, we apply temperature scaled softmax followed by KL divergence, as shown in Equation 9:


$$\begin{array}{l} \zeta_{C}=\frac{\varepsilon_{a}}{3}\overset{3}{\underset{a=1}{\sum }}\mathrm{KL}\left(\mathrm{softmax}\left(\frac{F_{\ell_{a}}^{T}}{\tau_{C}}\right)∥\mathrm{softmax}\left(\frac{F_{\ell_{a}}^{S}}{\tau_{C}}\right)\right) \end{array}$$ (9)

where ε_*a*_∈ℝ is a layer specific importance coefficient used to emphasize different feature map layers (*P*3, *P*4, and *P*5) during the channel semantic alignment process. Here, *a* = 1, 2, 3 correspond to the three feature maps at different spatial resolutions, and ℓ_*a*_ denotes the associated head layer in the YOLOv8 architecture. The feature tensor at layer ℓ_*a*_ is denoted as $F_{\ell_{a}}\in {\mathbb{R}}^{C_{\ell_{a}}\times H_{\ell_{a}}\times W_{\ell_{a}}}$. To improve alignment effectiveness, we assign larger weights to lower level features by setting ε_1_>ε_2_>ε_3_, which strengthens the alignment focus on *P*3 and enhances the detection of small objects commonly present in UAV imagery. The parameter τ_*C*_>0 represents the temperature scaling factor used in the channel semantic alignment loss.

**(5) Output block and unified objective** Based on the above calculations, the student is optimized with a composite loss, as shown in Equation 10:


$$\begin{array}{l} \zeta_{SD}=\zeta_{CE}+\lambda_{1}\zeta_{box}+\lambda_{2}\zeta_{G}+\lambda_{3}\zeta_{C} \end{array}$$ (10)

where ζ_*CE*_ is the standard classification loss, ζ_*box*_ is the bounding box regression term, and λ_1_, λ_2_, λ_3_≥0 weight the alignment losses. This joint objective enforces global consistency while retaining local personalization, thus alleviating overfitting and drift in heterogeneous federated environments.

#### 4.1.2 Drift compensation mechanism

In federated UAV object detection, the disparate distribution of images among devices results in incongruent local optimization goals, leading to divergent model update trajectories and subsequent model drift. To mitigate this issue, FL-JSDDC proposes the incorporation of a local drift variable for modeling and correction. Each client is tasked with managing a drift vector φ_*i*_ = *w*−*w*_*i*_ to monitor the variance between its local model and the prevailing global model, subsequently fine tuning the model parameters prior to aggregation. To provide an approximate assessment of the local model drift, a drift regularization term is introduced as follows:


$$\begin{array}{l} \psi^{i}=||\varphi_{i}+w_{i}-w|{|}^{2} \end{array}$$ (11)

Each client uses this regularization term and the empirical loss term on its corresponding dataset to train both the model parameters and the local drift variable, thus converting the constrained optimization problem into an unconstrained one.

In FL-JSDDC, the drift compensation loss for each client consists of two components: the drift regularization term and the gradient correction term. The drift regularization term is used to penalize the deviation of the local model from the global model, while the gradient correction term adjusts the local model toward the global model by correcting the local gradient during the training process, as shown in Equation 12:


$$\begin{array}{l} \zeta_{DC}=\frac{\alpha }{2}\psi^{i}\left(\varphi_{i};w_{i},w\right)+\xi_{i}\left(\varphi_{i};\mu_{i},\mu \right) \end{array}$$ (12)

where, α is the weight coefficient for the drift regularization term, and ξ_*i*_ controls the gradient correction term for stochastic gradient optimization. To smooth out gradient heterogeneity, we set the gradient correction term as $\xi_{i}\left(\varphi_{i};\mu_{i},\mu \right)=\frac{1}{\eta E}\langle \varphi_{i},\varphi_{i}-\varphi \rangle$, where η is the learning rate, and *E* is the number of training iterations in one round. μ_*i*_ is the local update of the *i*-th client's local parameters in the previous round, and μ is the average update of all clients' local parameters in the previous round. In the *t*-th round, we have: ${\mu_{i}}^{t}=w_{i}^{t}-w_{i}^{t-1}$ and $\mu^{t}=\frac{1}{N}\overset{N}{\underset{i=1}{\sum }}{\mu_{i}}^{t}$, where $w_{i}^{t}$ and $w_{i}^{t-1}$ are the local model parameters of client *i* in the *t*-th and (*t*−1)-th rounds, respectively. The purpose of this term is to reduce the variance of the local gradients.

Next, we introduce the update rule for the local drift variable φ_*i*_. Taking the *t*+1-th iteration as an example, in FL-JSDDC, the local drift variable tracks the gap between the local model and the global model. In the *t*+1-th round of training, we assume that the global model parameters are updated to *w*^*t*+1^, while the local model parameters remain fixed. Then we can update the local drift variable using $\varphi_{i}^{t+1}=\varphi_{i}^{t}+\left(w^{t+1}-w^{t}\right)$, but due to the unavailability of global data, it is impossible to directly update the global model.

Assume we first update the local model parameters from $w_{i}^{t}$ to $w_{i}^{t+1}$. Then we consider the following two points: 1) At the beginning of each round, the local model parameters are assigned to the global model parameters: $w_{i}^{t}=w^{t}$. 2) For client *i*, the local model parameters $w_{i}^{t+1}$ are an estimate of the updated global model parameters *w*^*t*+1^. Thus, we can approximate the update of the local drift variable as:


$$\begin{array}{l} \varphi_{i}^{t+1}=\varphi_{i}^{t}+\left(w^{t+1}-w^{t}\right)\approx \varphi_{i}^{t}+\left(w_{i}^{t+1}-w_{i}^{t}\right) \end{array}$$ (13)

**Figure 3:**
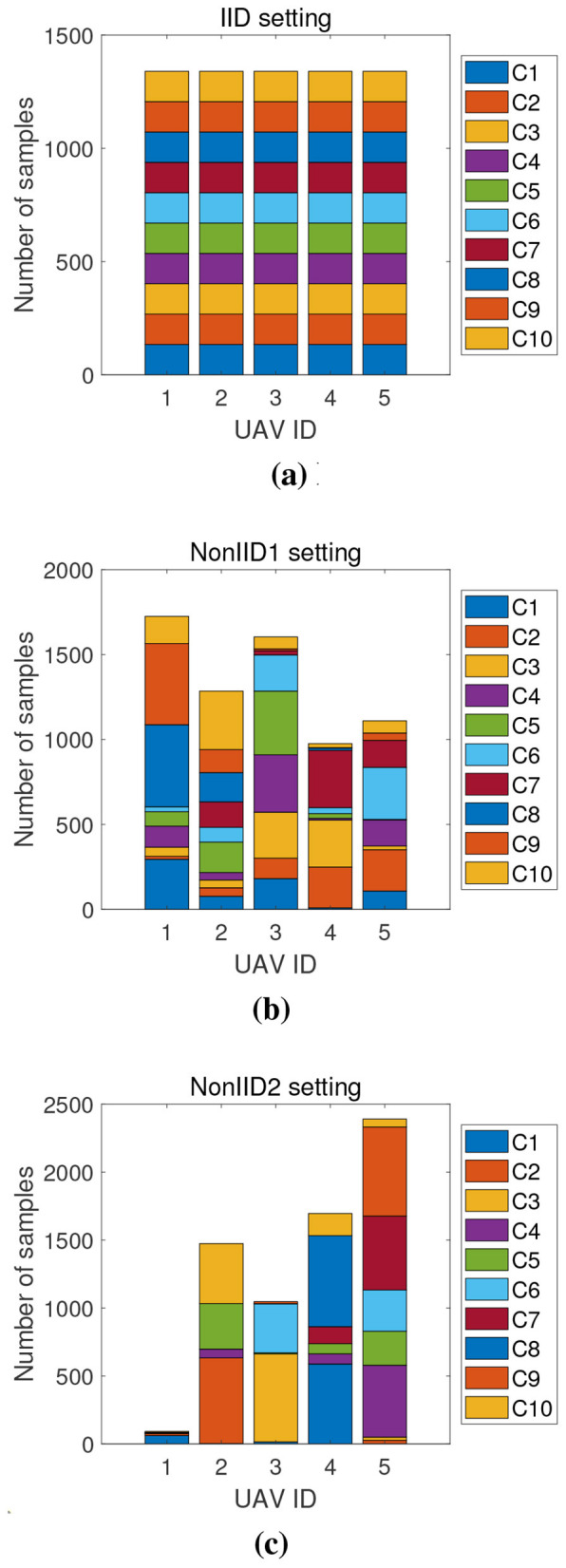
Simulated data distribution across UAV clients under three partitioning schemes. From left to right: **(a)** IID setting where class distributions are uniform across clients. **(b)** ND(1) setting with mild label imbalance; and **(c)** ND(2) setting exhibiting severe non-IID distributions generated via a Dirichlet process. C1–C10 denote the ten object categories in the VisDrone2019-DET dataset.

In this way, we use the update of the local model parameters to adjust the local drift variable (Algorithm 1 and Figure 3).

Algorithm 1 **FL-JSDDC: federated learning with joint self- distillation and drift compensation.**

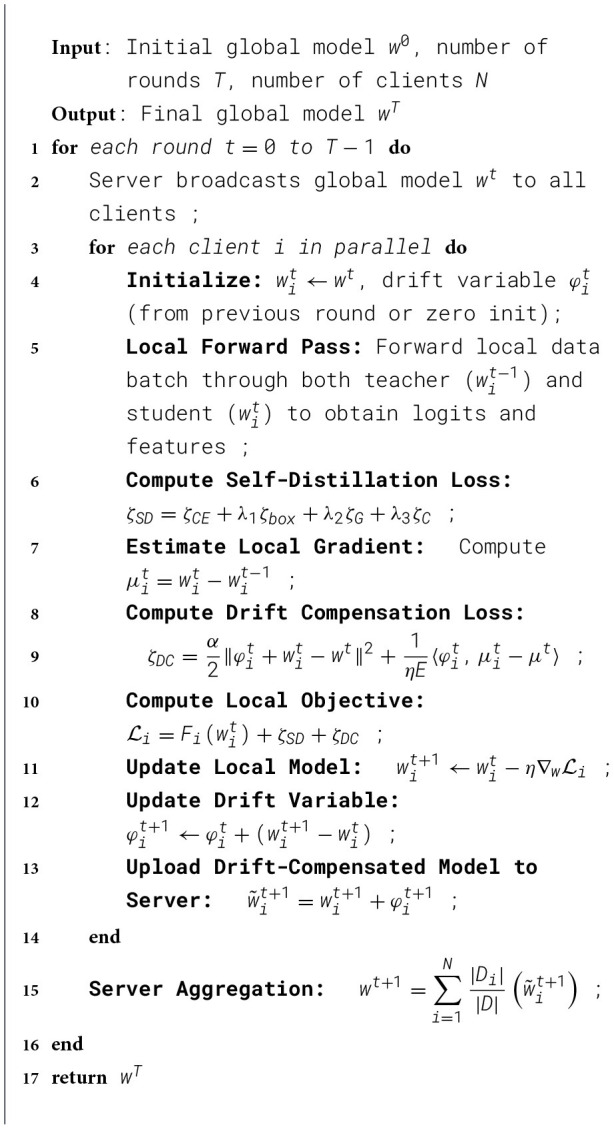

To update the global model parameters, each client uses its local drift variable $\left(\varphi_{i}^{t+1}+w_{i}^{t+1}\right)$ to correct the local model parameters before model aggregation. Then, each client uploads the corrected local parameters to the server. Similar to FedAvg, the server performs a weighted average of the corrected local parameters to obtain the global model parameters:


$$\begin{array}{l} w^{t+1}=\overset{N}{\underset{i=1}{\sum }}\frac{\left|D_{i}\right|}{\left|D\right|}\left(\varphi_{i}^{t+1}+w_{i}^{t+1}\right) \end{array}$$ (14)

where *D* is the global dataset. The final joint local objective function in FL-JSDDC is formulated as:


$$\begin{array}{l} \underset{w_{i}}{\min}\;F_{i}\left(w_{i}\right)+\zeta_{SD}+\zeta_{DC} \end{array}$$ (15)

By jointly integrating self-distillation and drift compensation, FL-JSDDC enhances both knowledge transfer and parameter alignment during local training. This leads to faster convergence and improved detection performance under non-IID data distributions.

### 4.2 Procedure of the proposed algorithm

In this section, we outline the key steps of the proposed FL-JSDDC algorithm, which integrates both self-distillation and drift compensation mechanisms to tackle data heterogeneity in federated object detection.

The algorithm proceeds through the following steps:

**(1) Initialization:** At the beginning of each communication round *t*, the server broadcasts the global model *w*^*t*^ to all clients. Each client *i* initializes its local model $w_{i}^{t}\leftarrow w^{t}$ and prepares its drift variable $\varphi_{i}^{t}$, which is either retained from the previous round or initialized to zero.
**(2) Local training:** Each client performs local training on its private dataset *D*_*i*_ using an objective that combines supervised detection loss, self-distillation loss (guided by the historical model $w_{i}^{t-1}$), and an drift compensation loss designed to address model drift.
**(3) Parameter update:** The client updates its local model parameters based on the total loss and adjusts the drift variable $\varphi_{i}^{t}$ to account for the parameter change since the previous round.
**(4) Transmission:** Each client uploads a drift compensated version of its updated model, denoted as ${\tilde{w}}_{i}^{t+1}$, which combines the local model and the corresponding drift variable.
**(5) Global aggregation:** The server aggregates the received drift compensated models from all clients using a weighted average to obtain the updated global model *w*^*t*+1^ for the next round.

The FL-JSDDC algorithm integrates client-specific features with global model alignment to enhance convergence speed and detection performance in non-IID environments. Through the incorporation of self-distillation and drift compensation, this algorithm establishes a resilient and effective federated UAV object detection framework in scenarios with non-IID data distributions.

The FL-JSDDC framework, as proposed, entails a moderate increase in computational load. Self-distillation necessitates an additional forward pass and auxiliary loss computations, resulting in a per-batch cost approximately 1.4-1.6 × higher than that of conventional YOLOv8n training. Conversely, drift compensation entails lightweight vector operations with minimal overhead. Both components operate exclusively on the client side, without augmenting communication requirements. The accelerated convergence of FL-JSDDC diminishes the total number of rounds, thereby counterbalancing the augmented local computational burden.

## 5 Experiments

In this section, we assess the efficacy of the FL-JSDDC method by contrasting it with various advanced FL approaches across diverse datasets and conditions. The assessment centers on two key metrics: (1) convergence rate and (2) model detection accuracy.

### 5.1 Datasets

Experiments were conducted using the VisDrone2019-DET dataset (Zhu et al., 2019), a prominent object detection dataset extensively applied in smart surveillance and autonomous driving contexts. The dataset comprises 10,209 images in total (6,471 training, 548 validation, and 3,190 test images), with over 540,000 annotated bounding boxes across 10 object categories: pedestrian, person, car, van, bus, truck, motor, bicycle, awning-tricycle, and tricycle. The dataset poses notable challenges due to dense object presence, intricate backgrounds, and occlusions, rendering it ideal for assessing detection accuracy in real world federated UAV object detection scenarios.

**Figure 4:**
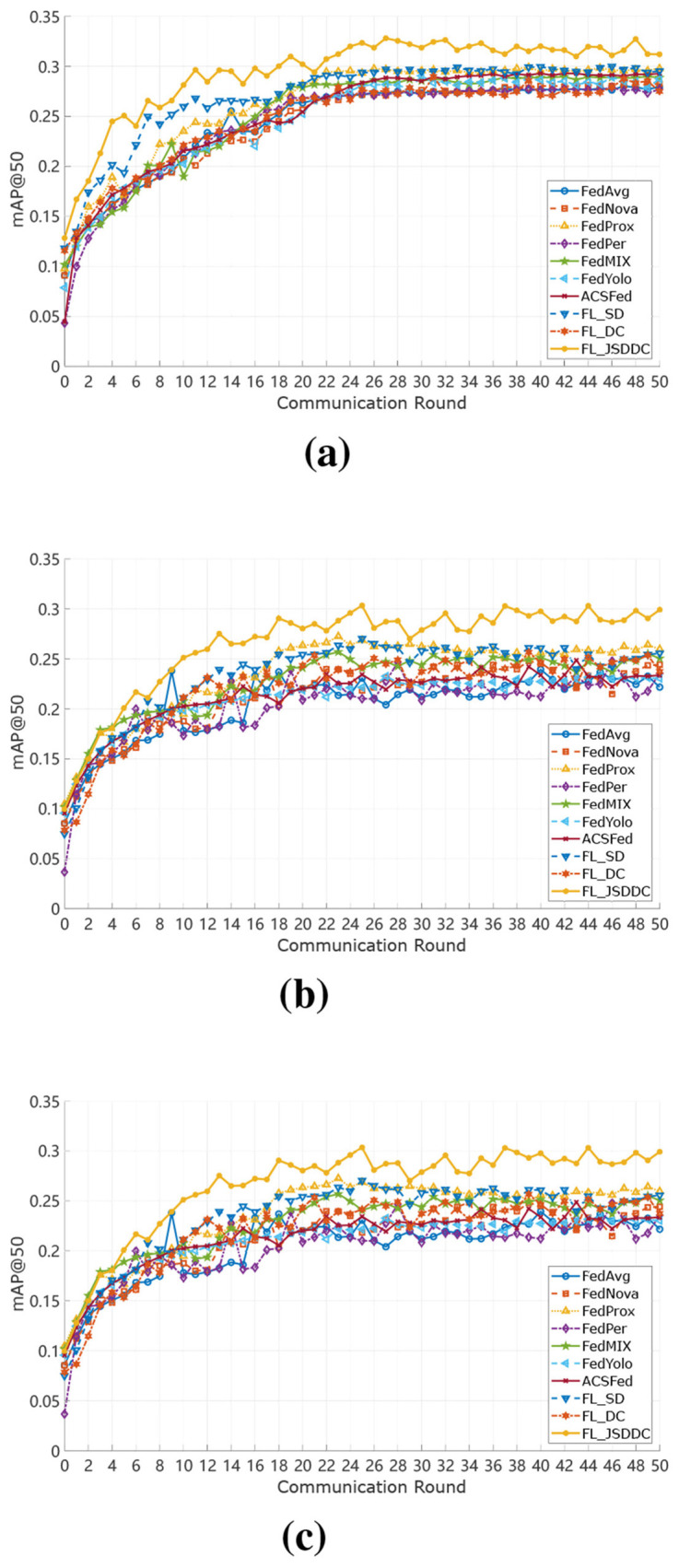
Detection Performance comparison of different FL algorithms over 50 communication rounds under **(a)** IID setting, **(b)** ND(1) setting, and **(c)** ND(2) setting. The proposed FL-JSDDC consistently outperforms baseline methods across all settings in both convergence speed and final detection performance.

To systematically evaluate the detection performance of various FL algorithms under different data heterogeneity scenarios, we construct three typical data partitioning schemes based on the VisDrone2019-DET dataset, as illustrated in Figure 4.

In the **IID setting**, all UAV clients sample data uniformly from the entire dataset, ensuring similar class distributions across clients. This setting approximates an ideal environment with independent and identically distributed (IID) data. In the **ND(1) setting**, we introduce moderate label imbalance by slightly altering the class distributions among clients. This simulates practical scenarios where different UAVs observe different patterns due to varying perspectives or environments. In the **ND(2) setting**, we employ a Dirichlet distribution with concentration parameter α = 0.3 to generate severely skewed class distributions. The small α value produces highly imbalanced allocations where some clients may have minimal or no samples for certain classes. This significantly increases the heterogeneity across clients, where each client may contain only a limited subset of classes or even a single class. Such a setting realistically reflects challenges like data silos and class absence in practical federated UAV object detection deployments. These partitioning schemes provide a comprehensive foundation for evaluating model robustness in increasingly heterogeneous federated environments.

### 5.2 Baselines

We conducted a comparative analysis of FL-JSDDC with various FL methodologies, such as FedAvg (Mehta and Aneja, 2024), FedNova (Wang et al., 2020), FedProx (Li et al., 2020), FedPer (Arivazhagan et al., 2019), FedYolo (Zhang et al., 2023), and ACSFed (Chen et al., 2022) in the context of federated learning. FedAvg employs weighted averaging of local updates to update the global model. FedNova introduces a normalization mechanism to mitigate local update bias in non-IID scenarios. FedProx incorporates a proximal regularization term to restrict local update deviation. FedPer enhances model robustness by refining the update process. In addition to classification tasks, we consider FedMIX (Yoon et al., 2021) as a benchmark for object detection in federated settings. FedYolo employs a modular architecture and pretrained transformer components to improve adaptation in federated settings, addressing inter-client variability through spatial-wise representation learning. ACSFed employs an Earth Mover's Distance-based method to evaluate client contributions and selectively engages clients to alleviate the effects of subpar or significantly divergent local updates. In contrast to FL-JSDDC, these strategies prioritize preserving model coherence between clients and the server, rather than directly addressing the challenge of local model deviation during parameter aggregation.

To further validate the robustness of our method, we conduct ablation studies by separately evaluating: FL-DC, which applies only drift compensation; FL-SD, which applies only self-distillation; and the full FL-JSDDC framework that combines both modules.

### 5.3 Implementation details

We adopt a standard FL architecture in our experiments. During each communication round, multiple clients perform local training on their private datasets and upload model updates to a central server for global aggregation. All experiments were conducted on a server with NVIDIA RTX 3090 GPU. Following the settings of the baseline methods (Mehta and Aneja, 2024; Li et al., 2020; Wang et al., 2020; Arivazhagan et al., 2019; Zhang et al., 2023; Chen et al., 2022; Yoon et al., 2021), we use the Adam optimizer for all local training, along with cosine annealing learning rate scheduling. For consistency across experiments, the batch size for local training is set to 32, the number of local training epochs is 15, and the initial learning rate is 0.001. These settings align with best practices in federated object detection tasks.

We employ the YOLOv8n variant as the backbone detector. To demonstrate deployment feasibility on real UAV platforms, we evaluate inference performance on an NVIDIA Jetson Nano (128-core Maxwell GPU, Quad-core ARM Cortex-A57 CPU, and 4 GB LPDDR4 memory), operating within 5–10 W and weighing about 300 g. This setup achieves over 20 FPS at 640 × 480 resolution, and fits UAVs with a payload capacity of 1–2 kg, such as the DJI Matrice 200/300 series, ensuring real time applicability under resource constraints.

For the proposed FL-JSDDC algorithm, the drift compensation coefficient is set to α = 0.08. The weights for self-distillation alignment losses are set as λ_1_ = 0.3 for bounding box alignment, λ_2_ = 0.3 for global class probability alignment, and λ_3_ = 0.4 for channel semantic alignment. The L2 regularization weight is set to β = 0.01. The number of participating clients is set to 5 across all experiments. For FedProx, the proximal regularization parameter μ is set to 0.1 for fair comparison. The hyperparameters of other baseline methods are kept consistent with those reported in their original publications.

### 5.4 Evaluation metrics

In this study, we adopt convergence speed and model detection performance under data heterogeneity as the primary evaluation metrics, which jointly reflect the robustness of the model. Specifically, convergence speed is measured by the number of communication rounds required to reach a target detection performance, to evaluate the overall detection performance of the model on object detection tasks, we adopt mean Average Precision at an IoU threshold of 0.5 (**mAP@50**) as the primary detection performance metric, as it reflects both classification correctness and localization precision under practical conditions.

Specifically, **mAP@50** is defined as the mean of Average Precision (AP) values computed across all object categories. For each category *c*∈{1, …, *C*}, the AP is obtained by integrating the precision recall curve:


$$\begin{array}{l} {\text{AP}}_{c}=\overset{1}{\underset{0}{\int }}{\text{P}}_{c}\left(r\right)\text{d}r, \end{array}$$ (16)

where P_*c*_(*r*) denotes the precision at recall level *r* for class *c*. A prediction is considered correct if the Intersection over Union (IoU) between the predicted bounding box and the ground truth box exceeds 0.5.

The final mAP@50 is computed by averaging over all *C* categories:


$$\begin{array}{l} \text{mAP@50}=\frac{1}{C}\overset{C}{\underset{c=1}{\sum }}{\text{AP}}_{c}. \end{array}$$ (17)

This robustness metric has gained increasing attention in recent FL research (FedTest, 2025; Hu et al., 2024). By considering both aspects, our robustness metric provides a comprehensive perspective for evaluating the robustness of each method under various data distribution scenarios. It enables deeper insights into how different algorithms cope with data heterogeneity and local model drift in federated object detection environment.

### 5.5 Evaluation tasks

In this study, we design a series of evaluation tasks to comprehensively assess the detection performance of FL algorithms under different data heterogeneity and client scale conditions. All experiments are conducted based on the VisDrone2019-DET dataset.

To simulate varying degrees of data heterogeneity, we consider three distinct distribution settings. In the uniform distribution scenario, each client receives data uniformly and randomly sampled from the entire dataset, ensuring that all clients share a similar class distribution. In the mildly non-IID scenario, the data is still randomly assigned, but the class distribution varies slightly across clients, which reflects practical cases where different clients may observe different data patterns. In the highly non-IID setting, the client data is partitioned using a Dirichlet distribution (Qu et al., 2022) to induce significant disparity in class proportions among clients, thereby emulating extreme heterogeneity in practical federated UAV object detection scenarios.

Furthermore, to evaluate model detection performance and convergence speed under different system scales, we conduct experiments involving 5, 15, and 35 participating clients under each of the above distribution scenarios. These tasks enable us to analyze the robustness of each algorithm in response to increasing data heterogeneity and client population, particularly focusing on how detection performance evolves as distribution skewness and the number of participants grow.

### 5.6 Results and analysis

We conduct extensive experiments to validate the advantages of FL-JSDDC in terms of convergence speed and model detection performance. The proposed method is evaluated under varying participation levels, client scales, and degrees of data heterogeneity to assess its robustness. All reported results represent the mean detection performance of local models evaluated on each client's respective test set, aggregated over 10 repeated experiments to ensure statistical reliability.

Since both FL-JSDDC and the baseline algorithms consume the same computational resources per communication round, the detection performance objectives of FL-JSDDC are twofold: 1) to accelerate model convergence and reduce communication overhead, and 2) to improve detection performance across diverse training environments. The results demonstrate that the robustness of FL-JSDDC consistently outperforms that of existing federated object detection optimization methods, as shown in Table 1.

**Table 1:** We denote the communication round of each method to achieve the target detection performance as (R#) and the corresponding convergence speedup relative to FedAvg as (S↑).

Model	5 Clients						15 Clients						35 Clients					
	ND(1)		ND(2)		IID		ND(1)		ND(2)		IID		ND(1)		ND(2)		IID	
	R#	S↑	R#	S↑	R#	S↑	R#	S↑	R#	S↑	R#	S↑	R#	S↑	R#	S↑	R#	S↑
Target: 21%																		
FedAvg	13	1	19	1	10	1	16	1	23	1	13	1	19	1	26	1	17	1
FedNova	15	0.86	24	0.79	13	0.77	19	0.84	-	-	15	0.87	23	0.83	-	-	19	0.89
FedProx	9	1.44	31	0.61	8	1.25	11	1.45	-	-	10	1.30	15	1.27	-	-	13	1.31
FedPer	13	1	25	0.76	11	0.91	14	1.14	30	0.77	12	0.87	23	0.74	34	0.76	23	0.74
FedMIX	11	1.18	28	0.68	10	1	13	1.23	-	-	11	1.18	16	1.19	-	-	22	0.77
FedYolo	11	1.18	21	0.90	12	0.83	15	1.07	24	0.96	12	1.08	19	1	28	0.93	16	1.06
ACSFed	10	1.30	23	0.83	12	0.83	13	1.23	26	0.88	11	1.18	17	1.12	31	0.84	14	1.21
FL-SD	10	1.30	22	0.86	9	1.11	12	1.33	24	0.96	10	1.30	15	1.27	27	0.96	14	1.21
FL-DC	12	1.08	27	0.70	11	0.91	14	1.14	29	0.79	12	1.08	18	1.06	-	-	17	1
FL-JSDDC	5	2.6	12	1.58	4	2.5	6	2.66	15	1.53	6	2.17	9	2.11	15	1.73	6	2.83

Methods that fail to reach the target are marked with “-”.

#### 5.6.1 Evaluation of FL-JSDDC on convergence speed

Table 1 compares the number of communication rounds (R#) required for FL-JSDDC and seven baseline methods (FedAvg, FedNova, FedProx, FedPer, FedMIX, FedYolo and ACSFed), along with two ablation variants, FL-SD and FL-DC to reach the target detection performance of 21%. All experiments are conducted on the VisDrone2019-DET dataset under three data distribution settings: **IID**, **ND(1)**(mild non-IID), and **ND(2)**(severe non-IID).

In the IID setting, data is uniformly distributed, and local datasets are similar across clients. As a result, the divergence between local and global models is minimal, yielding robust training. For instance, with 15 clients, FedAvg requires 13 rounds to reach the target detection performance due to its simple weighted averaging strategy, which fails to correct client model deviations. FedNova slightly improves convergence by normalizing updates, but still needs 15 rounds. FedProx introduces a proximal term to restrict update deviation, reducing the round count to 10, but inconsistencies in update directions remain. FedPer adjusts global updates for better robustness, yet converges in 12 rounds, on par with FedAvg. FedMIX leverages a mixing strategy to fuse local and global models, achieving modest improvement with 11 rounds. FedYolo and ACSFed achieve convergence in 12 and 11 rounds respectively under IID settings, but their advantages in generalization and adaptive selection are less pronounced due to the statistical similarity among clients.

The ablation study conducted by FL-SD focuses solely on self-distillation, demonstrating convergence within 10 rounds under IID settings, suggesting that incorporating soft guidance from the global model facilitates convergence. However, the absence of explicit constraints on model drift impedes FL-SD from achieving optimal speed. On the other hand, FL-DC, which exclusively implements drift compensation, achieves convergence in 12 rounds, effectively mitigating drift but lacking personalized feature learning. Integration of both mechanisms in FL-JSDDC yields superior detection performance, reaching the target in only 6 rounds. This outcome underscores the significance of combining self-distillation and drift compensation for rapid and robust convergence. A comparison between FL-SD and FL-JSDDC underscores the substantial enhancement in global alignment through drift correction, while the disparity between FL-DC and FL-JSDDC underscores the critical role of knowledge transfer in ensuring resilient representation learning.

To further validate the robustness of our method, we conduct ablation studies by separately evaluating: FL-DC, which applies only drift compensation; FL-SD, which applies only self-distillation; and the full FL-JSDDC framework that combines both modules.

In the context of ND(1) settings involving 15 participating clients exhibiting moderate heterogeneity, the convergence speed deteriorates across all baseline methods. Specifically, FedAvg, FedNova, and FedPer necessitate 16, 19, and 14 rounds, respectively, while FedProx and FedMIX demonstrate slightly improved performance, requiring 11 and 13 rounds, respectively. FedYolo and ACSFed converge in 15 and 13 rounds, respectively. The escalation in round requirements for convergence with even minor heterogeneity underscores the challenge. Notably, FL-SD and FL-DC exhibit comparable performance to the aforementioned baselines, suggesting limited efficacy of a singular approach. In contrast, the integration of FL-JSDDC, incorporating both modules, achieves notably enhanced convergence efficiency.

In the ND(2) scenario involving 15 client participants with diverse data distributions, convergence performance notably deteriorates across established methods. Specifically, both FedAvg and FedPer necessitate 23 and 30 communication rounds, respectively, to attain the desired detection performance level (21% mAP@50). FedYolo achieves convergence in 24 rounds, while ACSFed does so in 26 rounds. Conversely, FedNova, FedProx, and FedMIX fall short of meeting the performance target within the 50 round threshold, underscoring their vulnerability to data distribution imbalances. These findings underscore the substantial impediment posed by significant data heterogeneity on model aggregation and training resilience. Notably, FL-JSDDC accomplishes the performance goal in a mere 15 rounds, showcasing exceptional adaptability and robustness in non-IID settings.

The detection performance is influenced by the scale of clients. A smaller number of clients, such as 5, leads to less fragmented data, lower heterogeneity, and quicker convergence across all methods. Notably, FL-JSDDC demonstrates the fastest convergence under these circumstances. Conversely, with 35 clients, data fragmentation increases, leading to higher heterogeneity and decreased convergence efficiency. Most baseline methods experience notable slowdowns, particularly FedAvg and FedNova. Even in this challenging scenario, FL-JSDDC maintains its superior speed and robustness. This resilience is attributed to its design, wherein self-distillation allows clients to extract globally aligned features from limited local data, and drift compensation regulates local deviations from the global model. These mechanisms enable FL-JSDDC to uphold synchronization and robust convergence, even in demanding conditions characterized by a large number of clients and significant data heterogeneity.

#### 5.6.2 Evaluation of FL-JSDDC on detection performance

Table 2 reports the best detection performance achieved by FL-JSDDC under various settings and compares it against multiple baseline methods. Experiments are conducted on the VisDrone2019-DET dataset across different client scales (5, 15, 35) and data distribution settings (IID, ND(1), ND(2)).

**Table 2:** Final detection performance (mAP@50) of different algorithms under various client scales and data heterogeneity settings.

Model	5 Clients			15 Clients			35 Clients		
	ND(1)	ND(2)	IID	ND(1)	ND(2)	IID	ND(1)	ND(2)	IID
FedAvg	23.21%	21.17%	28.44%	22.18%	19.51%	26.71%	18.64%	16.23%	24.32%
FedNova	24.90%	21.51%	29.12%	24.43%	19.35%	27.24%	19.73%	15.02%	25.49%
FedProx	25.17%	22.32%	29.94%	23.97%	18.50%	27.79%	21.01%	14.94%	25.18%
FedPer	24.91%	21.35%	28.12%	23.56%	19.22%	27.68%	20.55%	15.76%	25.05%
FedMIX	25.84%	21.27%	29.32%	25.05%	19.43%	27.53%	20.72%	16.98%	25.28%
FedYolo	23.84%	20.83%	28.72%	22.12%	19.58%	26.83%	19.21%	16.44%	24.76%
ACSFed	23.37%	21.71%	29.34%	22.04%	19.96%	27.10%	19.92%	16.41%	25.01%
FL-SD	25.57%	22.16%	29.78%	25.56%	20.06%	28.21%	21.18%	17.75%	26.52%
FL-DC	23.47%	21.91%	28.94%	23.75%	19.18%	26.48%	18.33%	15.76%	25.03%
FL-JSDDC	29.15%	24.91%	32.61%	27.92%	21.29%	30.19%	25.69%	20.27%	28.49%

In the IID scenario with uniformly distributed client data, model convergence exhibits greater resilience and leads to enhanced overall detection performance. For example, when considering 5 clients, FL-JSDDC demonstrates an mAP@50 of 32.61%, showcasing a significant improvement over FedAvg (28.44%), and FedProx (29.94%). Additionally, FedYolo and ACSFed achieve 28.72% and 29.34%, respectively. As the client count increases, FL-JSDDC sustains strong detection performance, achieving 28.49% with 35 clients, surpassing FedAvg (24.32%) and FedNova (25.49%). These results underscore not only the algorithm's robustness under balanced data distributions but also its scalability.

In the ND(1) scenario involving 5 participating clients with moderate data heterogeneity, conventional methods exhibit significant performance deterioration. Specifically, FedAvg and FedNova experience a decrease in mAP@50 to 23.21% and 24.90%, respectively, while FedYolo and ACSFed achieve 23.84% and 23.37%, respectively, showing a further decline as the number of clients increases. In contrast, FL-JSDDC demonstrates consistent detection performance, attaining 29.15% with 5 clients and 27.92% with 15 clients. These findings suggest that FL-JSDDC effectively mitigates the effects of mild data heterogeneity and sustains robust detection performance across varying numbers of clients.

In the ND(2) scenario characterized by heightened heterogeneity, conventional methods exhibit notable declines in detection accuracy. Specifically, when dealing with 35 clients, FedAvg and FedNova achieve only 16.23% and 15.02% accuracy, while FedProx and FedPer drop to 14.94% and 15.76%, respectively. In contrast, FedYolo and ACSFed attain 16.44% and 16.41% accuracy, respectively, in the same setting. Despite these formidable challenges, FL-JSDDC stands out by achieving 20.27% accuracy in this extreme scenario, surpassing all baseline methods and showcasing remarkable resilience to highly non-IID conditions.

From the perspective of client scale, increasing the number of clients from 5 to 35 leads to more fragmented data and greater training difficulty. While most baseline methods suffer significant detection performance degradation, FL-JSDDC maintains a high level of detection performance. For instance, FL-JSDDC achieves 24.91% mAP@50 with 5 clients and still retains 20.27% with 35 clients, markedly outperforming all other methods. This result further validates its ability to cope with the increased heterogeneity introduced by larger client populations.

Overall, FL-JSDDC consistently delivers superior detection performance across all data partition settings and client scales. This improvement is primarily attributed to the synergistic effects of its self-distillation and drift compensation mechanisms. The self-distillation component guides local models to learn globally consistent features, improving their adaptability. Meanwhile, the drift compensation component effectively suppresses excessive local model drift, enhancing the robustness of global aggregation. Together, these mechanisms enable FL-JSDDC to maintain high detection performance and robust convergence even in challenging federated environments, making it a promising solution to the client drift problem in federated object detection.

To further demonstrate the convergence behavior and robustness of the proposed method, Figure 4 presents the detection performance curves of FL-JSDDC and baseline methods over 50 communication rounds under three data heterogeneity settings. All results are obtained under a federated configuration involving 5 participating clients. In the IID setting (Figure 4a), where client data distributions are balanced, FL-JSDDC rapidly achieves high detection performance, outperforming other methods from the early stages of training.

Under the ND(1) setting (Figure 4b), where client distributions slightly differ, FL-JSDDC still maintains a clear advantage in both convergence speed and robustness, while FedAvg and FedNova exhibit slower and more volatile trends.

In the most challenging ND(2) scenario (Figure 4c), where class imbalance across clients is severe, baseline methods suffer significant detection performance degradation and stagnation. However, FL-JSDDC demonstrates strong resilience, achieving higher detection performance with faster convergence, validating the robustness of its joint self-distillation and drift compensation design in addressing model drift and local overfitting in non-IID environments.

### 5.7 Discussion

In summarizing the simulation outcomes, FL-JSDDC exhibits distinct advantages in convergence speed and detection performance across varied data heterogeneity scenarios. In comparison to standard methods, FL-JSDDC not only attains quicker convergence (e.g., achieving target detection performance within 3-5 rounds) but also sustains notably superior detection performance amidst escalating client numbers or data imbalance levels.

The enhancements primarily stem from the synergistic operation of two key modules: the self-distillation mechanism enhances the preservation of personalized local knowledge while conforming to global semantics, thereby enhancing adaptability. Simultaneously, the drift compensation mechanism adeptly mitigates local model drift, ensuring a more stable global aggregation. This dual framework demonstrates notable resilience in highly non-IID scenarios, where conventional federated object detection methods experience sluggish convergence and diminished detection performance.

Moreover, as the number of clients increases from 5 to 35, FL-JSDDC maintains consistently high detection performance, confirming its scalability. These results underscore the potential of the proposed framework for practical implementation in distributed UAV networks, where client diversity and limited resources are prevalent.

## 6 Conclusion

In this study, we introduce FL-JSDDC, a federated UAV object detection framework designed for non-IID data scenarios. Through the incorporation of self-distillation and drift compensation mechanisms, FL-JSDDC enhances model detection accuracy, convergence rate, and performance on decentralized client devices. Empirical findings demonstrate the superior performance of FL-JSDDC compared to current federated learning approaches, particularly in non-IID settings. Subsequent research endeavors will concentrate on customizing FL-JSDDC for dynamic client engagement and investigating model compression methods to facilitate its implementation in resource limited environments.

## Data Availability

The original contributions presented in the study are included in the article/supplementary material, further inquiries can be directed to the corresponding author.
